# Adaptation and psychometric properties of the life conditions scale in university staff: a study in Spain and Colombia

**DOI:** 10.3389/fpubh.2026.1945598

**Published:** 2026-10-20

**Authors:** Beatriz Sánchez-Hernando, Patricia Marín-Maicas, Jorge Casaña-Mohedo, Ana Pellín-Carcelén, Luís López-Pérez, Emmanuel Echaniz-Serrano, Raúl Juárez-Vela, Michal Czapla, Pablo Roche-Soguero, Nohora Dulfay Bohórquez-Martínez, Marisol Urrego-Aguilera, Vicente Gea-Caballero

**Affiliations:** 1 Faculty of Health Science, International University of Valencia, Valencia, Spain; 2 Research Group in Community Health and Care SALCOM, International University of Valencia, Valencia, Spain; 3 Sapienf (B53_23R) Research Group, Department of Physiatry and Nursing, Faculty of Health Sciences, University of Zaragoza, Zaragoza, Spain; 4 Health Center “Valdespartera-Montecanal”, Aragón Health Care System (SALUD), Zaragoza, Spain; 5 Faculty of Nursing and Podiatry, University of Valencia, Valencia, Spain; 6 Department of Physiatry and Nursing, University of Zaragoza, Zaragoza, Spain; 7 GRUPAC Research Group, Department of Nursing, University of La Rioja, Logroño, La Rioja, Spain; 8 Instituto de Neurociencias de Castilla y León (INCYL), Lab-12, Universidad de Salamanca, Instituto de Investigación Biomédica de Salamanca (IBSAL), Calle Pintor Fernando Gallego, Salamanca, Spain; 9 Division of Scientific Research and Innovation in Emergency Medical Service, Department of Emergency Medical Service, Faculty of Nursing and Midwifery, Wroclaw Medical University, Wroclaw, Poland; 10 Faculty of Health Science, Corporación Universitaria Iberoamericana IBERO, Bogotá, Colombia

**Keywords:** health promotion, lifestyle, mental health, psychometrics, surveys and questionnaires, universities

## Abstract

**Background:**

Health promotion within the university setting requires valid and reliable instruments to identify factors related to lifestyle, emotional well-being and mental health among staff. Although the Life Conditions Scale (LifeConScale) had previously been validated in adolescents, there were no instruments specifically integrating these dimensions for university personnel. Therefore, adaptation and validation for use with higher education professionals is necessary.

**Objective:**

To adapt the LifeConScale and examine its psychometric properties in university staff, including Teaching and Research Staff and Technical, Management, Administrative and Services Staff.

**Materials and methods:**

A cross-sectional study was conducted with 440 professionals from two universities in Spain and Colombia. The instrument was adapted via a Delphi process with health and health-promotion experts. Common-factor exploratory factor analysis with parallel analysis and oblique rotation assessed internal structure; Kaiser-Meyer-Olkin and Bartlett’s test evaluated sampling adequacy. Reliability was estimated with McDonald’s omega. Measurement invariance for core dimensions (personal wellbeing, anxiety, physical activity) between countries was examined through nested multigroup confirmatory factor analysis (configural, metric and scalar).

**Results:**

Sampling adequacy was adequate (KMO = 0.850; *p* < 0.001). Parallel analysis and common-factor exploratory factor analysis with oblique rotation identified seven interpretable factors: personal wellbeing, anxiety, physical activity, snacking, leisure screen use, social media use, and fruit and vegetable intake. The Personal Wellbeing Index and GAD-7 showed no cross-loadings and a moderate latent correlation, supporting discriminant validity; the AUDIT-C was retained as an independent validated scale scored with its original algorithm. Reliability (McDonald’s omega) was excellent for anxiety (ω = 0.929), wellbeing (ω = 0.922) and social media (ω = 0.920), and good for physical activity (ω = 0.859), leisure screens (ω = 0.795), fruit and vegetables (ω = 0.760) and snacking (ω = 0.753). Heterogeneous dietary and sleep indicators were treated as formative and not aggregated into summative scores.

## 1 Introduction

Health is a multidimensional construct that integrates physical, psychological and social wellbeing, moving beyond the traditional conception focused solely on the absence of disease. Health is understood as a state of wellbeing that enables individuals to respond appropriately to environmental demands (1, 2). This perspective has driven the development of health promotion strategies aimed at acting on determinants that condition individual and community wellbeing. The United Nations recognizes health as a fundamental right and includes it among the Sustainable Development Goals (SDG 3: Good Health and Well-being) (3, 4).

Health promotion is defined as the process that enables people to increase control over the determinants of their health and to improve it (2, 5). Because the environments where people live, study and work directly influence health-related habits and behaviours, universities are recognized as strategic contexts for health promotion actions (6, 7). The “Health Promoting Universities” movement integrates health promotion principles into institutional culture, policies and actions to improve quality of life and wellbeing across the academic community (6, 8, 9). University settings, beyond their role in research and training, are living environments where environmental, organizational and personal factors interact and affect the health and wellbeing of all members (7, 10, 11). As such, universities are ideal, low-cost settings to foster healthy lifestyles (6–8, 12, 13).

Implementing health promotion strategies in universities requires prior knowledge of the characteristics and needs of the target population. A rigorous health diagnosis is essential to identify risk and protective factors related to lifestyle, emotional wellbeing and life skills. Healthy diet, regular physical activity, substance use prevention and adoption of safe behaviours are fundamental to maintaining adequate health (1, 7, 9, 14). Available evidence shows a progressive loss of healthy habits, with dietary patterns diverging from nutritional recommendations and increased sedentary behaviour associated with digital transformation (15, 16). Reliable information is therefore needed to guide effective health promotion interventions.

Within the university context, this need is particularly relevant for staff. Both Teaching and Research Staff (TRS) and Technical, Management, Administrative and Services Staff (TMAS) may be exposed to factors affecting health and wellbeing, such as high work demands, academic and administrative responsibilities, occupational stress and sedentary behaviours. Early identification of these factors via health diagnostics enables preventive actions, supports institutional occupational health policies and promotes healthier work environments. Staff wellbeing can also impact institutional outcomes such as educational quality, organizational climate, process efficiency and sense of belonging (12, 13, 17). For these reasons, valid and reliable instruments that jointly assess lifestyle, emotional wellbeing and life skills in university professionals are needed. The LifeConScale (18) was originally developed to integrate these components in adolescents; this study aims to adapt and validate it for university staff to provide a robust tool for health diagnostics and to inform health promotion strategies.

## 2 Materials and methods

### 2.1 Design

An observational, descriptive, cross-sectional study was conducted during the 2025–26 academic year. The study setting comprised two higher education institutions: the International University of Valencia (VIU), Spain, and the Corporación Universitaria Iberoamericana, Colombia.

### 2.2 Population and sample

The target population included all professionals employed at both institutions, encompassing Teaching and Research Staff (TRS) and Technical, Management, Administrative and Services Staff (TMAS). The combined workforce of the two universities was approximately 2,300 people. A minimum sample size of 330 participants was estimated to ensure adequate representativeness (95% confidence interval, 5% margin of error, 50% heterogeneity). All eligible staff were invited to participate; no probabilistic sampling technique was applied. Inclusion criteria: active employment at either university during 2025–26 and voluntary completion of the questionnaire. Exclusion criteria: staff on leave, sick leave or any circumstance preventing normal job performance at data collection. Recruitment was coordinated with Human Resources via internal communication platforms and the corporate Microsoft Teams account. Participation was voluntary and anonymous.

### 2.3 Data collection instrument and study variables

The questionnaire was based on the LifeConScale (18), originally validated in adolescents, with adaptations for the professional context. Three validated scales were incorporated: AUDIT-C for alcohol consumption (3 items, Likert 0–4; validated in Spain) (19), the Personal Wellbeing Index (PWI; Likert 0–10) (20) for emotional wellbeing cross-culturally adapted and validated for the Spanish population (21, 22), and the GAD-7 for generalized anxiety (7 items scored 0–3; total 0–21; Spanish cut-offs: mild 6–9, moderate 10–14, severe 15–21) (23). The adaptation process was conducted between March and April 2025 using the Delphi method (24) with a panel of nine experts over two rounds (24). Expert inclusion criteria: recognized health sciences professional (nursing, medicine, psychology, psychiatry), ≥5 years’ experience in health and mental health promotion, and experience in instrument validation and diagnostic use.

Study variables were grouped into sociodemographic and occupational variables (age, sex, university, staff category—TRS or TMAS—faculty/unit, work modality—onsite or virtual—work schedule—full-time or part-time—height, weight, calculated body mass index, and self-perceived health) and health variables (dietary habits, sleep pattern, physical activity, screen use, alcohol consumption, anxiety level and subjective wellbeing).

### 2.4 Data analysis

Following the original tool’s procedure (18), descriptive analyses characterized the sample. Internal structure was examined via exploratory factor analysis using common-factor extraction (maximum likelihood) on the correlation matrix. Data adequacy was assessed with the KMO index (threshold > 0.75) and Bartlett’s sphericity test. The number of factors to retain was determined by parallel analysis (500 random datasets, 95th percentile criterion), rather than by the Kaiser criterion alone. An oblique (Promax) rotation was applied to allow for correlated factors, and the inter-factor correlation matrix was inspected to confirm its appropriateness. Loadings ≥ 0.40 were considered relevant. Reliability was estimated with McDonald’s omega and Cronbach’s alpha, with 95% bootstrap confidence intervals. Heterogeneous indicators not representing a common latent construct were treated as formative and not aggregated into summative scores. Because the sample came from Spain and Colombia, measurement invariance across countries was examined for the three core reflective dimensions (personal wellbeing, anxiety and physical activity) through a sequence of nested multigroup confirmatory factor models (configural, metric and scalar), evaluated with the change in CFI (ΔCFI ≤ 0.010) and RMSEA (ΔRMSEA ≤ 0.015) (25, 26). Missing data were handled by listwise deletion. Because missing values were concentrated in the GAD-7 items, the multigroup invariance analyses of the three core dimensions were conducted on the 406 participants with complete data on those items (102 from Spain and 304 from Colombia). Analyses were performed in Python 3.12 (pandas, numpy, factor_analyzer) and replicated in JASP 0.96, yielding concordant results. The exploratory factor analysis was conducted on the 402 participants with complete data on all analysed items, whereas the invariance analyses used the 406 participants with complete GAD-7 data.

### 2.5 Ethical considerations

The study followed the Declaration of Helsinki. The questionnaire was anonymous and data were treated confidentially in accordance with Spanish Organic Law 3/2018 and EU Regulation 2016/679. Authors declared no conflicts of interest or financial compensation. Participation was voluntary and uncompensated. The research project was approved by the Ethics Committee for Research and Teaching (CEID) of the International University of Valencia (code “CEID2025_07”).

## 3 Results

### 3.1 Sample Characteristics

The sample comprised 440 participants (66.1% women), mean age 38.05 years (SD = 11.79; range 18–79). 75.7% belonged to the Corporación Universitaria Iberoamericana and 24.3% to the International University of Valencia (VIU). 72.5% were TRS and 27.5% TMAS. Regarding BMI, 47.1% were normal weight, 38.6% overweight and 11.8% obese. Self-perceived health averaged 7.97 (SD = 1.36) on a 0–10 scale; 88.0% rated their health as good or excellent. Full sociodemographic details are presented in Table 1.

**Table 1:** Sociodemographic and health characteristics of the sample (*n* = 440).

Variable	Category/Metrics	*n* (%) or Mean (SD) [Range]
University	Corp. Universitaria Iberoamericana International Univ. of Valencia (VIU)	333 (75.7%) 107 (24.3%)
Sex	Female Male Prefer not to say	291 (66.1%) 147 (33.4%) 2 (0.5%)
Age (years)	Mean (SD) [Range]	38.05 (11.79) [18–79]
Residence	Urban Rural	390 (88.6%) 50 (11.4%)
Height (cm)	Mean (SD) [Range]	165.26 (8.97) [148–197]
Weight (kg)	Mean (SD) [Range]	69.13 (13.30) [42–125]
BMI (kg/m^2^)	Mean (SD) Normal Overweight Obese Underweight	25.24 (4.14) 204 (47.1%) 167 (38.6%) 51 (11.8%) 11 (2.5%)
Self-Perceived Health	Excellent Good Fair Poor Score (0–10 scale): Mean (SD)	90 (20.5%) 297 (67.5%) 49 (11.1%) 4 (0.9%) 7.97 (1.36)
Job Position	Faculty (TRS) Support Staff (TMAS)	319 (72.5%) 121 (27.5%)
Work Modality	On-site Remote	240 (54.5%) 200 (45.5%)
Commitment	Full-time Part-time	292 (66.4%) 148 (33.6%)

### 3.2 Data adequacy for exploratory factor analysis

The Kaiser-Meyer-Olkin (KMO) indicated satisfactory sampling adequacy (KMO = 0.850), comparable to that reported in the original adolescent validation (0.812). Bartlett’s test was significant (χ^2^ = 7770.8; df = 703; *p* < 0.001), supporting factorability.

Parallel analysis retained seven factors; the additional components suggested by the Kaiser criterion fell below the 95th percentile of random eigenvalues and were not retained. The common-factor solution with oblique (Promax) rotation explained 54.3% of shared variance. The resulting factor structure and the rotated loadings for each item are presented in Table 2, and the parallel-analysis scree plot is shown in Figure 1.

**Table 2:** Rotated factor loadings (common-factor EFA, Promax rotation) and McDonald’s omega by factor.

Factor/Item	Loading	ω
1. Personal wellbeing (PWI)	—	0.922
Achievements	0.887	
Safety	0.872	
Future security	0.851	
Life satisfaction	0.850	
Community	0.794	
Relationships	0.778	
Spiritual life	0.631	
Health	0.597	
2. Anxiety (GAD-7)	—	0.929
GAD-7 item 4	0.869	
GAD-7 item 1	0.856	
GAD-7 item 2	0.851	
GAD-7 item 3	0.851	
GAD-7 item 5	0.822	
GAD-7 item 7	0.768	
GAD-7 item 6	0.733	
3. Physical activity	—	0.859
Vigorous physical activity	0.840	
Physical activity frequency	0.789	
Moderate physical activity	0.786	
Muscle strengthening	0.733	
4. Snacking	—	0.753
Sugar-sweetened beverages	0.811	
Salty snacks	0.801	
Sweets	0.653	
5. Leisure screen time	—	0.795
Leisure screens (weekends)	1.151	
Leisure screens (weekdays)	0.648	
6. Fruit and vegetables	—	0.760
Fruit	0.824	
Vegetables	0.815	
7. Social media use	—	0.920
Social media (weekdays)	0.989	
Social media (weekends)	0.787	
Formative/independent indicators (not aggregated)	—	—
Sleep duration	—	—
Cereals	—	—
Dairy	—	—
Meat	—	—
Breakfast (weekends)	—	—
AUDIT-C item 1*	—	—
AUDIT-C item 2*	—	—
AUDIT-C item 3*	—	—

Common-factor EFA (maximum likelihood) with oblique Promax rotation; *n* = 402. Loadings ≥ 0.40 shown. ω = McDonald’s omega. Em dashes (—) indicate not applicable: sleep and the listed dietary items did not load saliently on any factor and are treated as formative indicators. *AUDIT-C is retained as an independent validated scale (ω = 0.643) scored with its original algorithm.

**Figure 1:**
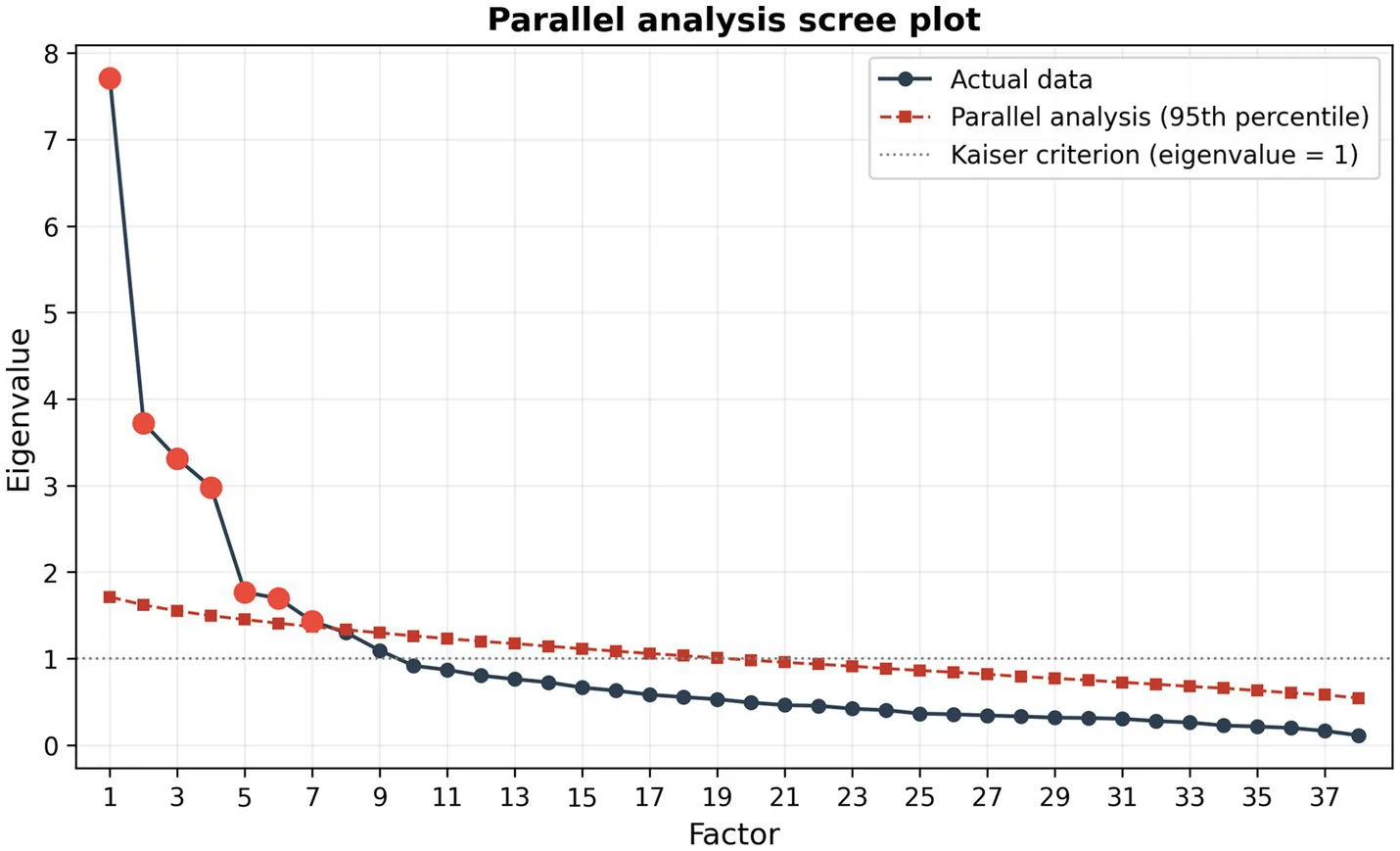
Parallel analysis. Observed eigenvalues (solid line) plotted against the 95th percentile of eigenvalues from 500 randomly generated data matrices (dashed line). Seven factors were retained, corresponding to the point at which the observed eigenvalues fall below the random threshold.

The seven factors were interpreted as: (1) personal wellbeing (PWI items); (2) anxiety (GAD-7 items); (3) physical activity; (4) snacking (sweets, salty snacks and sugar-sweetened beverages); (5) leisure screen use; (6) fruit and vegetable intake; and (7) social media use. The inter-factor correlation matrix showed associations up to r = 0.45, confirming the appropriateness of the oblique rotation. The AUDIT-C items did not form an emergent factor and were retained as an independent validated scale; the sleep item and several dietary items (cereals, dairy, meat, weekend breakfast) did not load saliently on any factor and were treated as formative indicators. Rotated factor loadings and reliability by factor are shown in Table 2.

Key loadings and reliability per factor were: personal wellbeing (PWI)—eight items, loadings 0.597–0.887, ω = 0.922; anxiety (GAD-7)—seven items, loadings 0.733–0.869, ω = 0.929; physical activity—four items, loadings 0.733–0.840, ω = 0.859; snacking—three items, loadings 0.653–0.811, ω = 0.753; social media—two items, ω = 0.920; leisure screens—two items, ω = 0.795; fruit and vegetables—two items, loadings 0.815–0.824, ω = 0.760. The AUDIT-C, scored as an independent validated scale, showed ω = 0.643, consistent with its brevity (three items). Omega equalled or exceeded Cronbach’s alpha for every reflective block.

Most items loaded > 0.60 on their respective factors, except the sleep hours item (loading = 0.378), below the conventional 0.40 threshold. The two breakfast-frequency items were coded so that higher scores indicate more frequent breakfast; their negative loadings (−0.447 and −0.357) on the snacking component reflect the expected inverse association between snacking and regular breakfast, and no items were reverse-coded prior to reliability estimation.

### 3.3 Internal consistency

Reliability of the reflective blocks, estimated with McDonald’s omega and 95% bootstrap confidence intervals, was good to excellent: anxiety (ω = 0.929), wellbeing (ω = 0.922), social media (ω = 0.920), physical activity (ω = 0.859 [0.834, 0.881]), leisure screens (ω = 0.795), fruit and vegetables (ω = 0.760) and snacking (ω = 0.753 [0.699, 0.799]). Discriminant validity was supported by the absence of cross-loadings between the personal wellbeing and anxiety factors and their moderate latent correlation (r = −0.37). The AUDIT-C was retained as an independent validated scale rather than as an emergent factor, as its independence is guaranteed by design. Heterogeneous dietary and sleep indicators, which do not represent a common latent construct, were treated as formative and were not combined into summative scales.

### 3.4 Measurement invariance across countries

Multigroup confirmatory factor analysis was conducted to test the cross-national equivalence of the three core reflective dimensions (Personal Wellbeing, Anxiety, and Physical Activity) between the Spanish (*n* = 102) and Colombian (*n* = 304) subsamples with complete GAD-7 data (*n* = 406). Following the sequential procedure recommended by Chen (25), three nested models were estimated: a configural model (equal form), a metric model (equal factor loadings), and a scalar model (equal loadings and intercepts). Invariance at each step was evaluated using the change in comparative fit index (ΔCFI ≤ 0.010) and the change in root mean square error of approximation (ΔRMSEA ≤ 0.015), which are less sensitive to sample size than the χ^2^ difference test (25, 26).

The configural model showed adequate fit (CFI = 0.952, RMSEA = 0.063, SRMR = 0.051), supporting the same three-factor structure in both countries. Constraining the factor loadings to equality (metric model) did not degrade fit; model fit remained essentially identical (CFI = 0.953, RMSEA = 0.061, SRMR = 0.054), with ΔCFI = +0.001 and ΔRMSEA = −0.002, both well within the recommended thresholds. Imposing equal intercepts (scalar model) likewise preserved fit (CFI = 0.946, RMSEA = 0.064, SRMR = 0.054), with ΔCFI = −0.007 and ΔRMSEA = +0.003. Full scalar invariance was therefore supported, indicating that both the factor loadings and the item intercepts of the three core dimensions are equivalent across the Spanish and Colombian samples. Fit indices for the three models are reported in Table 3.

**Table 3:** Measurement invariance of the three core dimensions across Spain and Colombia.

Model	CFI	TLI	RMSEA	RMSEA 90% CI	SRMR	ΔCFI	ΔRMSEA	Decision
Configural	0.952	0.945	0.063	[0.054, 0.071]	0.051	—	—	—
Metric (loadings)	0.953	0.949	0.061	[0.052, 0.069]	0.054	+0.001	−0.002	Invariance supported
Scalar (loadings + intercepts)	0.946	0.944	0.064	[0.055, 0.072]	0.054	−0.007	+0.003	Invariance supported

Estimator = ML. ΔCFI and ΔRMSEA computed relative to the preceding model. Criteria (25): ΔCFI ≤ 0.010 and ΔRMSEA ≤ 0.015.

Because full scalar invariance held, latent means were freely estimated in the Colombian group with Spain as the reference. Relative to the Spanish sample, the Colombian sample showed a higher latent mean on Personal Wellbeing (0.285, *p* = 0.034) and a lower latent mean on Physical Activity (−0.491, *p* < 0.001), while the latent mean difference in Anxiety did not reach significance (−0.167, *p* = 0.052).

## 4 Discussion

This study aimed to validate the LifeConScale (18) in a sample of university professionals, adapting an instrument originally developed for adolescents. Results support the instrument’s suitability for this new population, while revealing particularities in its internal structure that warrant discussion.

Sampling adequacy was satisfactory (KMO = 0.850), comparable to the adolescent validation, and Bartlett’s test confirmed the appropriateness of factor analysis. The seven-factor common-factor solution explained 54.3% of shared variance; because common-factor analysis models shared rather than total variance, this figure is not directly comparable to the total variance reported under principal component analysis and reflects the more conservative and appropriate estimation of latent structure.

### 4.1 Factor structure and differences from adolescent population

A notable finding was fragmentation of the dietary dimension. Whereas in adolescents dietary habits tended to cluster more cohesively, in adult professionals dietary items did not form a single cohesive dimension: snacking and fruit-and-vegetable intake emerged as distinct reflective factors, while the remaining indicators (cereals, dairy, meat, weekend breakfast) did not load on any common factor and were treated as formative. This fragmentation aligns with theoretical expectations given adult consumption patterns: dietary decisions in adulthood are more differentiated and respond to distinct logics (convenience, available time, entrenched habits, specific food preferences) compared to a more homogeneous adolescent pattern influenced by family and school contexts (18, 24). Thus, rather than indicating a limitation, this differentiation reflects the greater complexity of adult dietary behaviours.

### 4.2 Reliability of components

Core dimensions showed high reliability (ω between 0.753 and 0.929), consistent with the robustness of the incorporated validated scales (PWI, GAD-7) and the physical activity block. Discriminant validity was supported by the clean separation of the wellbeing and anxiety factors, which showed no cross-loadings and only a moderate latent correlation. The AUDIT-C, as a brief validated scale, was administered and scored independently; its independence from the remaining dimensions is established by design rather than by its emergence as a factor, and its items did not load on any other dimension.

Under the earlier principal-component specification, several food-related components fell below the 0.70 threshold. When the snacking items were correctly modelled as a three-item reflective block in the common-factor solution, however, reliability reached an acceptable level (ω = 0.753 [0.699, 0.799]), indicating that the previously low value largely reflected model misspecification and the sensitivity of Cronbach’s alpha to the number of items (27–29). By contrast, the remaining dietary indicators (cereals, dairy, meat, and weekend breakfast) did not load saliently on any common factor, consistent with their measuring heterogeneous behaviours that may coexist independently rather than a homogeneous latent construct (e.g., consuming sweets does not necessarily imply skipping breakfast). These indicators are therefore more formative than reflective; Cronbach’s alpha, designed for reflective indicators, is not the appropriate reliability statistic for them (28, 30, 31). Consequently, these indicators were not treated as summative scales but retained as individual diagnostic indicators of specific dietary habits.

Similarly, the sleep hours item showed a weak loading (0.378) on the physical activity component. Although sleep and physical activity are theoretically related and central to an active, healthy lifestyle, the weak loading indicates sleep shares only partial variance with that dimension (32). Sleep hours should therefore be treated as a complementary indicator within the health diagnosis rather than as an integral part of the physical activity measurement.

### 4.3 Cross-national invariance

The establishment of full scalar invariance for the three core reflective dimensions across the Spanish and Colombian samples is a central strength of the present study and directly addresses the requirement that cross-cultural equivalence be demonstrated through formal multigroup confirmatory factor analysis rather than through congruence coefficients alone (25, 26). Whereas the Tucker congruence coefficient establishes only structural (configural) similarity, the nested-model approach adopted here provides a considerably stronger test: metric invariance confirms that the items are related to their underlying constructs with the same strength in both countries, and scalar invariance further confirms that respondents from Spain and Colombia who share the same latent standing endorse the items at the same level. Notably, model fit did not deteriorate, and for the metric model even improved marginally, as equality constraints were added, a pattern indicative of robust cross-national stability of the wellbeing, anxiety, and physical-activity dimensions.

The practical implication is important: because scalar invariance was supported, the observed latent-mean differences between the two countries can be interpreted as genuine differences in the constructs themselves rather than as artifacts of differential item functioning or culturally divergent response styles. The higher Personal Wellbeing and lower Physical Activity means observed in the Colombian sample can thus be regarded as substantively meaningful. This licenses the direct comparison of scale scores across the two national contexts and supports the use of the adapted LifeConScale in cross-national research on the living and working conditions of university staff.

### 4.4 Limitations

Limitations include focus on internal structure and reliability without assessing criterion validity (e.g., correlations with absenteeism, productivity or objective health records) or convergent validity with other occupational wellbeing instruments-, areas for future research. Test–retest reliability was not examined, leaving temporal stability unassessed. Unequal sample distribution between countries and the relatively small Spanish subsample limited invariance analyses to the most robust theoretical dimensions. The cross-sectional design precludes causal inference.

## 5 Conclusion

The adapted LifeConScale is an appropriate instrument for health diagnostics among university professionals, showing satisfactory sampling adequacy and a seven-factor structure identified through common-factor analysis and parallel analysis. Core reflective dimensions, personal wellbeing, anxiety, physical activity, snacking, screen use and fruit and vegetable intake, exhibited good to excellent reliability, and the validated scales behaved as well-differentiated dimensions, supporting discriminant validity. Measurement equivalence between Spanish and Colombian contexts for central dimensions supports cross-cultural applicability.

The heterogeneous dietary indicators that did not load on any common factor (cereals, dairy, meat, and weekend breakfast) showed formative rather than reflective characteristics; therefore, it is recommended not to aggregate them into a summative score but to interpret their items as individual dietary indicators. The snacking and fruit-and-vegetable factors, by contrast, behaved as coherent reflective dimensions and can be scored as such. The sleep hours item should be treated as a complementary indicator. Overall, the tool provides a solid basis for comprehensive health diagnostics in university settings and informs the design, implementation and evaluation of staff-focused health promotion strategies. Future studies should address criterion and convergent validity and test–retest reliability to consolidate psychometric properties.

## Data Availability

The datasets presented in this article are not publicly available due to institutional restrictions imposed by the participating university. Requests to access the datasets should be directed to the corresponding author and will be considered subject to the prior authorization of the participating university, in accordance with its data access policies. Requests to access the datasets should be directed to beatriz.sanchez0@professor.universidadviu.com.
